# Clear cell gynecologic carcinomas: about 5 cases

**DOI:** 10.11604/pamj.2019.34.87.18505

**Published:** 2019-10-14

**Authors:** Bergaoui Haifa, Zouari Ines, Njima Manel, Daldoul Amira, Zaied Sonia, Njim Laila, Mhabrech Houda, Hadded Anis, Felah Raja

**Affiliations:** 1 Department of Gynecology and Obstetrics, El Omrane Hospital of Monastir, Monastir, Tunisia; 2 Department of Pathological Anatomy, Fattouma Bourguiba University Hospital of Monastir, Monastir, Tunisia; 3 Department of Medical Oncology, Fattouma Bourguiba University Hospital of Monastir, Monastir, Tunisia; 4 Department of Radiology, Regional Hospital of Ksar Hellal, Monastir, Tunisia

**Keywords:** Clear cell carcinoma, gynecologic carcinoma, surgery, chemotherapy

## Abstract

Clear cell carcinoma (CCC) can simulate yolk sac tumor if the location is ovarian. In this case, the morphological distinction between these tumors is often difficult, but immunohistochemistry, the determination of CA125, of alpha fetoprotein (AFP) and the response to chemotherapy are particularly useful for solving this differential diagnosis problem. Endometrial and vaginal localization is even rare and appears to be related to distilbene uptake for vaginal localization. Whatever the gynecological location, CCC seems to have a poor prognosis. We report 5 cases of gynecological CCC including 1 case of vaginal carcinoma, 1 case of endometrial carcinoma and 3 cases of ovarian carcinoma. The definitive pathological examination concluded with the CCC diagnosis for all cases. Our purpose is to report these rare cases, their diagnosis, prognosis and therapeutic management.

## Introduction

Clear cell carcinoma (CCC) is a rare pathological entity in gynecology. CCC accounts for approximately 5% of ovarian carcinomas and few cases of other gynecological localization have been reported in the literature. Thus, the diagnosis is based essentially on the final pathological examination and is sometimes difficult requiring the use of immunohistochemistry. The therapeutic management joins the protocol of gynecological cancers [1]. In the localized stages, clear cell histology appears to be a factor of poor prognosis. In this context, the absence of reliable prospective series does not allow the realization of a conservative treatment for ovarian adenocarcinoma. These data and the fact that the benefit of chemotherapy was not influenced by the histological type in the ICON1 and ACTION studies led to the proposal of adjuvant chemotherapy in all CCC at any stage. For endometrial or vaginal localization, additional radiotherapy depends on the stage. The surveillance consists essentially of a clinical and para-clinical examination. For the ovary, a dosage of CA 125 markers every 3 months for 2 years then every 6 months until the 5^th^ year, then once a year. If the markers were initially normal, it is necessary to perform an endo-vaginal ultrasound every 3 to 6 months [2]. Through our work we plan to emphasize on the characteristics, treatment and prognosis of this rare entity.

## Methods

This was a retrospective study covering the period from January 2012 to December 2017. It included 5 patients treated for gynecologic CCC in the Maternity Center of Monastir.

## Results

### Observation 1

**Figure 1:**
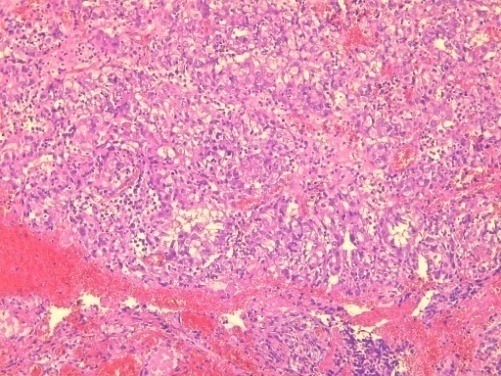
Solid sheet of tumor cells (HEx40)

**Figure 2:**
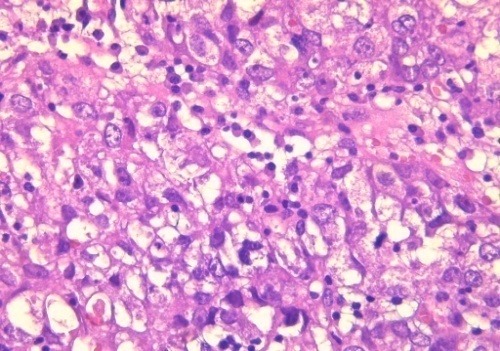
Solid sheet of tumor cells with voluminous clear cytoplasm and pleomorphic nuclei (HEx400)

Young girl H.Z. 23 years old with no pathological or familial history of neoplasia, consulted for recurrent bleeding. General examination was without peculiarity, gynecological examination found minimal red bleeding exteriorized by vagina, the hymen was intact and the vulva was without abnormality. Ultrasound showed small uterus, thin endometrium and appendages without abnormalities. Patient underwent hormonal treatment with an appointment for control in 3 months. Due to the persistence of metrorrhagia, a magnetic resonance imaging (MRI) was requested and identified vaginal mass of 3 cm, the rest of the examination was without particularity. The patient underwent vaginoscopy which revealed a presence of a 3 cm ulcerated mass. Resection was performed and histopathological examination revealed: CCC of the cervico-vaginal area (Figure 1, Figure 2). The patient was taken to another gynecological department where vaginoscopy was resurfaced and biopsies revealed that the tumor was resected in its entirety and a complement of laparoscopic pelvic and lumbo-aortic dissection was done. Lymph node involvement makes the indication of complementary radiotherapy.

### Observation 2

**Figure 3:**
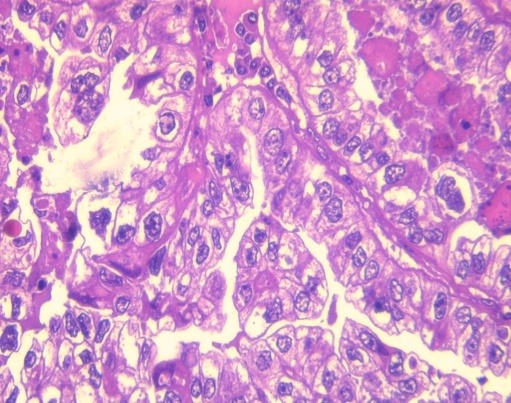
Infiltration of the uterine wall by a tumor proliferation with solid and tubuloglandular growth (HEx40)

**Figure 4:**
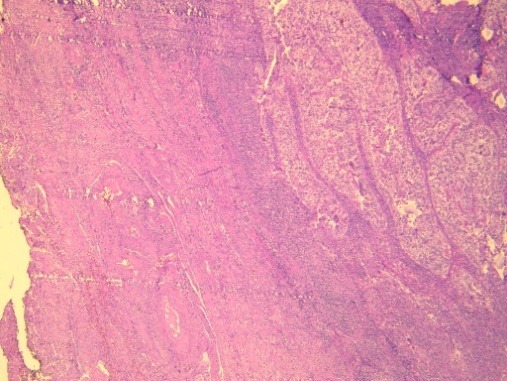
Both clear and hobnail cells are present with nuclear pleomorphism (HEx400)

Mrs M.A. aged 80 years was multipare, menopausal since the age of 52, cachectic and had hypertension under medical treatment, was followed for a month in the gastric department for epigastralgia. She underwent a fibroscopy and a biopsy which revealed a congestive gastro-bulbitis with histopathological diagnosis of chronic gastritis. In the course of the exploration of weight loss and postmenopausal bleeding, the patient underwent a gynecological examination which revealed a large friable tumor of the cervix bleeding on contact. The endometrial tissue fragment was sent for anatomopathological examination and the diagnosis was CCC of the endometrium (Figure 3, Figure 4). An ultrasound complement showed a tumor process that occupies the entire uterine cavity. An MRI showed voluminous isthmic mass delivered by the cervix, heterogeneous thick endometrium, myometrial infiltration lower than 50%. The tumor was classified IA. The patient was operated on and the final pathological examination showed CCC, tumor is involved in the isthmus with massive infiltration of the cervix, the most infiltrating part of the tumor was 1 mm from the serosa at the anterior isthmic. An additional radiotherapy has been indicated.

### Observation 3

Mrs C. 49-year-old (gravida 5, para 4), not yet menopausal with history of cholecystectomy and appendectomy and who is followed for hypertension under medical treatment. The patient consulted for pelvic pain. On examination, we found an increased abdominal and pelvic volume. Ultrasound confirms the clinical examination data and showed a 14 cm cystic and solid right pelvic mass. Computed tomography (CT) showed voluminous abdominopelvic mass at the expense of the right ovary of 16*12*11 cm with infiltration of the pelvic fat and a with ascites. Her serum CA125 level was elevated (162 U/mL). Scanned-guided biopsy was made and showed a low grade adenocarcinomatous proliferation of the ovary. Right ovarian mass with suspicious solido-cystic appearance invading the epiploon with anterior parietal implantation associated with right iliac adenopathy and ascites of moderate abundance were noted on MRI. After the completion of chemotherapy, the patient underwent a total abdominal hysterectomy and bilateral salpingo-oophorectomy, total omentectomy, bilateral pelvic lymph node dissection with definitive pathological examination concluded to a CCC of the ovary, the left appendix, the uterus and the epiploon. Additional adjuvant chemotherapy has been indicated.

### Observation 4

Mrs B.T. aged 51, (gravida1, para1), has secondary infertility of 21 years, was menopausal for 2 years, consulted for abdominopelvic pain and vertigo. Left lateral uterine mass of 11*09*08 cm solid-cystic was identified on ultrasound. CT revealed an irregular heterogeneous tumoral process with double component occupying the pelvis of 12*11*11 cm at the expense of the ovary. The patient underwent an exploratory laparotomy which confirmed the left ovarian mass suspected. The extemporaneous anatomopathological examination revealed an ovarian adenocarcinoma. The patient underwent radical treatment with definitive pathological examination of CCC of the left ovary without surface, uterus and omentum involvement. Adjuvant chemotherapy was done.

### Observation 5

Miss A.S. 39 years old with no particular past medical history, consulted for the discovery of bilateral ovarian cysts following an MRI done in the context of persistent low back pain. A pelvic examination identified a very large mass which was hardly movable. MRI showed solidocystic multilocular bilateral ovarian masses with poorly limited solid component and without lymphadenopathy. Thoraco-pelvic abdominal CT scan found 10 cm latero-uterine tumor masses of ovarian origin without pulmonary, hepatic and bone secondary lesions. The patient underwent laparotomy which revealed ovarian bilateral CCC with endometriotic lesions and left-sided borderline endometriotic tumor. The patient will benefit from adjuvant chemotherapy.

## Discussion

Gynecologic CCC is a rare disease. Its prevalence is one per 100,000 individuals and represents less than 5% of ovarian cancers and less than 1% of all gynecological malignancies [3]. Age at diagnosis varies by location. In our observation the age varies from 23 years to 80 years. The etiology of CCC is not well understood, seems to be progressive according to the histology of endometrial carcinoma and is confused with genital cord carcinomas for ovarian cancer [4, 5]. A recent study identified precursor lesions in 90% of cases of a woman with clear cell endometrial cancer, the lesions were usually isolated glands or surface epithelium in an endometrial region, otherwise she had a cytoplasm normal clear and/or eosinophilic with varying degrees of nuclear atypia [6]. Women with a family history of gynecologic cancer do not appear to be a risk factor for developing CCC. The risk factors for this type of carcinoma, as well as screening and diagnosis, are similar to those corresponding to each gynecological location of the cancer. For endometrial CCC, it appears that diagnosis can be made following an abnormal smear. Although smear is not a screening method for endometrial cancer, it tends to be abnormal in women with clear cell endometrial cancer [7-9]. The diagnostic means of reference remains the biopsy of the endometrium with a sensitivity greater than 99%. The CCC of the vagina is a rare disease affecting preferentially young women exposed in utero to distilbene. Currently, just over 700 cases have been described worldwide [10].

### Diagnosis and histology

The diagnosis of the histological type is done on surgical specimen or on biopsies and the final report confirms the diagnosis of the type CCC. Ovarian CCC is a histological subtype of epithelial disease. Most CCCs are unilateral. Typically, the surface section of the tumor reveals a unilocular cyst with one or more solid, yellow nodules protruding into the cyst. Cysts may contain “aqueous”, chocolate colored, mucinous or brownish liquids. Multilocular cysts are less common and occasional tumors are predominantly solid. The average size of CCC is 15 cm. It presents a distinct clinical behavior. There are marked geographic differences in the prevalence of CCC. CCC is more likely to be detected at an early stage compared to high grade serous cancers. Nevertheless, the responses remain well below those of other types of epithelial cancer. CCC of the endometrium has no particular macroscopic features. Histologically, it can have any of the following characteristics: papillary, tubulocystic or solid forms. These features may exist alone or in combination. The papillary form seems to be the most common [3]. Common features include intraluminal mucin, focal presence of intracytoplasmic vacuoles containing eosinophilic mucin, hyaline droplets and stromal hyalinization. The tumor should comprise more than 50% clear cell histology so that it can be described as CCC (by agreement of the Gynecologic Oncology Group (GOG) Pathology Committee). The vaginal CCC typically occurs at an early stage (90%) in young women (median: 19 years) with in utero exposure to distilbene reported in 60 to 70% of cases [11, 12]. Oncogenic Human Papillomavirus (HPV) or mutation of the P53 gene would play a minor role in the genesis of non-distilbene induced CCC [10].

### Prognosis

Clear cell carcinoma appears to have a more negative prognosis in ovarian carcinomas compared to other epithelial carcinomas. When confined to the ovary, the prognosis is good. However, if the disease is advanced, it is of very poor prognosis and resistance to the standard of treatment. The impact of clear cell histology compared to other types of histologic endometrial cancer is controversial. Two recent studies demonstrate that there is no difference in survival with stage I and II. However, survival was affected by the International Federation of Gynecology and Obstetrics (FIGO) stage compared to the endometrioid histological type [13]. Other studies suggest that the prognosis is more negative for clear cell histology compared to FIGO grade 3 in cancer of the liver, endometrioid endometrium. In a second study, clear cell histology was more likely to exhibit deep myometrial and lymphovascular invasion and ectopic disease compared to endometrioid endometrial cancer [14]. Histology of clear cells is an independent predictor of poor prognosis [15]. CCC of the vagina would have worse prognosis and higher risk of metastases. This cancer is not compatible with the maintenance of fertility [10].

### Treatment

Appropriate surgical treatment, followed by systemic chemotherapy, is recommended as initial therapy for patients with ovarian CCC. The standard surgical treatment for CCC patients is the same as for other epithelial ovarian cancers and includes hysterectomy, bilateral salpingo-oophorectomy, omentectomy, pelvic and para-aortic lymphadenectomy, and cytoreductive surgery for more advanced stages. The recommended regimen for postoperative chemotherapy is paclitaxel (175 mg/m^2^) combined with carboplatin (AUC 5-7.5), administered every 3 weeks for 6 cycles. Clear cell endometrial cancer treatment includes surgery, chemotherapy and/or radiotherapy, often in a multimodal combination. However, because of the rarity of this cancer, there are no trials evaluating these treatments in a study population comprising only women with clear cell endometrial cancer [16]. The classic treatment of vaginal locations is colpectomy in small volume forms of the lower vagina or enlarged colpohysterectomy in other forms. Radiation therapy is also indicated, either associated with localized tumor excision or brachytherapy. The results of these therapeutic options are comparable in terms of survival. In the early stages, in the absence of uterine malformation and after ovarian transposition, pregnancy may be considered. Treatment of advanced stages typically consists of exclusive radiotherapy [10].

## Conclusion

Clear cell tumors are an intriguing histological type of ovarian epithelial cancers, but also other gynecological sites with unique clinical features. Therapeutic management is similar to that of other histological types. Additional in vitro studies using CCC cell lines can identify the mechanism of chemoresistance in these tumors for ovarian localizations.

### What is known about this topic

- Clear cell carcinoma is a rare pathological entity in gynecology;
- The absence of prospective studies makes the therapeutic strategy non-consensual.

### What this study adds

- Through our work, we emphasize on the characteristics of Tunisian patients who had gynecologic clear cell carcinoma;
- This study points out the peculiarities of treatment and prognosis of gynecologic clear cell carcinoma among Tunisian patients.

## Competing interests

The authors declare no competing interests.
